# Is Concha Bullosa Associated with Nasal Septum Deviation and Mucosal Thickening of the Maxillary Sinuses? A Cone Beam Computed Tomography Study

**DOI:** 10.1055/s-0045-1809647

**Published:** 2025-09-10

**Authors:** Débora Costa Ruiz, Letícia de Andrade Souza, Amanda Farias-Gomes, Deborah Queiroz Freitas

**Affiliations:** 1 Department of Oral Diagnosis, Piracicaba Dental School, Universidade de Campinas, Piracicaba, SP, Brazil

**Keywords:** concha bullosa, nasal septum deviation, maxillary sinus, diagnostic imaging, cone-beam computed tomography

## Abstract

**Introduction:**

Concha bullosa (CB) is an anatomical variation that can affect respiratory function. Therefore, identifying it is important.

**Objective:**

To evaluate the prevalence of CB and its association with the side of pneumatization (right/left), nasal septum deviation (NSD), and mucosal thickening of the maxillary sinuses using cone-beam computed tomography (CBCT) exams from a Brazilian subpopulation. Also, to investigate the association between CB, sex and age.

**Methods:**

There were 297 CBCT exams assessed by two examiners who evaluated the presence of CB (> 50% pneumatization of the turbinate's height), NSD (deviation point of the nasal septum and crista galli angle >9 ^o^ ), and mucosal thickening of maxillary sinuses (mucosa around the sinus walls > 3 mm in height). The association between CB and side, NSD, mucosal thickening of the maxillary sinuses, sex, and age were evaluated by the chi-squared and Fisher's exact tests (α = 5%).

**Results:**

A total of 208 CB was found, corresponding to 11.7% of the evaluated turbinates, being more prevalent in the middle one ( *p* < 0.0001). Considering all exams, 132 (44.4%) had at least one CB. There was an association between the presence of middle left CB with NSD ( *p* = 0.028). No other significant associations were found ( *p* > 0.05).

**Conclusion:**

Almost half of the CBCT exams had at least one CB. Its prevalence was higher in the middle turbinate. There seems to be an association between middle CB and NSD. As for the other variables, no strong association was found.

## Introduction

Turbinates are paired structures located on the lateral walls of the nasal cavity, which act in filtration, humidification, and thermoregulation of the inhaled air. 1 2 They are divided into upper, middle, and lower turbinates. 3 4 The upper and middle turbinates are part of the ethmoid bone, and the lower ones are independent. 5 The nasal septum is a structure composed of bone and cartilage, located in the medial wall of the nasal cavity, which contributes to the proper functioning of the respiratory system. 6

Both structures may exhibit anatomical variations. 7 The nasal turbinates may be pneumatized (i.e. invaginated ethmoid cells filled with air), in which case they are referred to as concha bullosa (CB). 8 Nasal septum deviation (NSD) may be present, characterized by a misalignment of the septum relative to the midline. 8 9 10 A previous study showed that the presence of CB is not indicative of a cause or consequence for NSD. 4 However, other studies have concluded that there is a strong association between the presence of CB and NSD. 10 11

These anatomical variations are often asymptomatic. 8 12 However, in some cases, CB and NSD can be associated with dysregulated respiratory systems, which result in sinusitis, migraines, and ethmoiditis. 11 13 14 Under these conditions, there may be mucosal thickening of the maxillary sinuses. 10 15 It may be considered a pathological condition that requires adequate treatment when exceeding 3 mm. 15 Furthermore, in symptomatic cases, surgical intervention is often necessary to promote remission of symptoms and restore individuals' quality of life. 3 7

Endoscopy combined with complementary imaging exams contribute to the identification of these anatomical variations and to planning of the surgical procedure. 16 Computed tomography (CT) was once considered the complementary exam of choice for this evaluation but, recently, cone-beam computed tomography (CBCT) has proved to be useful for this task. 15 17

According to the literature consulted, there are no studies associating the three types of CB (upper, middle, and lower) with NSD or mucosal thickening of the maxillary sinus. Considering the impact of CB on the functioning of the respiratory system, especially in symptomatic individuals who require surgical intervention, the objective in the present study was to evaluate the prevalence and association of CB with the side of pneumatization (right/left turbinate), NSD, and mucosal thickening of the maxillary sinus, using CBCT exams from a Brazilian subpopulation. Furthermore, it was evaluated whether there is an association between the presence of CB and sex and age.

## Methods

This retrospective and cross-sectional study was initiated after approval by the local institutional review board (IRB) under the protocol number: 60637922.5.0000.5418.

### Sample Selection

There were 300 CBCT exams used in the present study, all obtained between January 2014 and December 2016. These exams are part of the database of the Oral Radiology clinic at a Dental School and were acquired using an i-CAT Next Generation device (Imaging Sciences International) for clinical reasons unrelated to the present research.

The inclusion criteria were exams acquired under the parameters of 5 mA, 120 kVp, 17.3 seconds of exposure time, voxel size of 0.3 mm ^3^ , and field of view of 23 ×17 cm. The exclusion criteria were patients under 18-years-old, those with dentomaxillofacial traumas or syndromes, and exams exhibiting motion artifacts that could impair the evaluation. Afterwards, 297 CBCT exams were included in the study: 142 males (18–64-years-old, mean: 32.04 ± 12.48 years), and 154 females (18–66-years-old, mean: 30.87 ± 11.47 years).

### Sample Assessment

All CBCT exams were exported in Digital Imaging and Communications in Medicine (DICOM) file format and assessed using the OnDemand3D (Cybermed, Irvine, CA, USA) software. Two dentomaxillofacial radiologists (3–5 years of experience) evaluated and recorded, in consensus, the presence of CB, NSD, and mucosal thickening in the maxillary sinuses. In cases of disagreement, a third observer (15 years of experience) was consulted. All evaluations were performed using a MDRC-2124 high-resolution medical display (Barco N.V.). The assessment of each variable was as follows:

#### Concha Bullosa

**Fig. 1:**
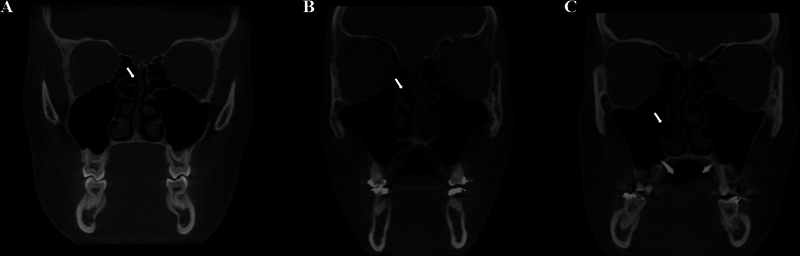
Coronal reconstructions of CBCT exams showing concha bullosa in the three turbinates. ( **A** ) The upper; ( **B** ) middle; and ( **C** ) lower concha bullosa. Arrows pointing to the hypodense area indicate the pneumatization of the turbinates.

The CBCT reconstructions of the upper, middle, and lower turbinates were assessed for the presence of CB. This anatomic variation was considered present when more than 50% of the vertical height (measured from superior to inferior in the coronal reconstruction) of the assessed turbinate was pneumatized ( Fig. 1 ). 10 In cases of bilateral CB (right and left pneumatized turbinates), both were recorded.

#### Nasal Septum Deviation

**Fig. 2:**
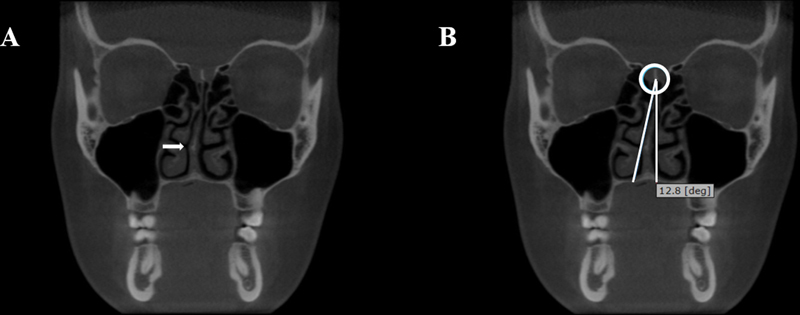
Coronal reconstructions of a CBCT exam showing a deviated nasal septum. ( **A** ) nasal septum deviated to the right. ( **B** ) The > 9 ^o^ angle formed by the most prominent deviation point of the nasal septum and the crista galli. Arrow pointing to the deviation.

For the evaluation of the nasal septum, the angle formed between the most prominent deviation point of the nasal septum and the crista galli was measured on a CBCT coronal reconstruction. If this angle was moderate (>9°), the NSD was considered present ( Fig. 2 ). 15 18

#### Mucosal Thickening of Maxillary Sinuses

**Fig. 3:**
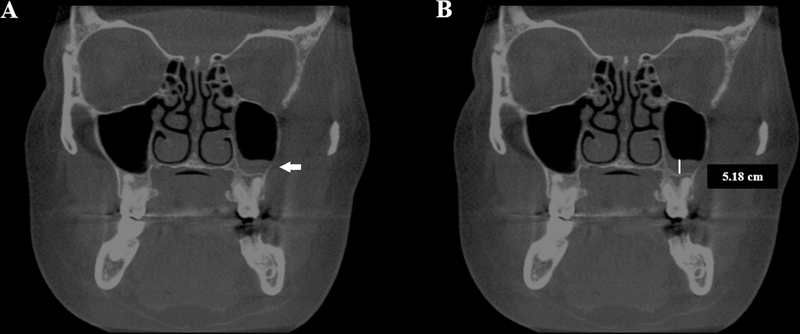
Coronal reconstructions of a CBCT exam showing the mucosal thickening of a maxillary sinus. ( **A** ) mucosal thickening that affects the left maxillary sinus. ( **B** ) mucosal thickening of the maxillary sinus with more than 3 mm of height. Arrow pointing to the mucosal thickening.

The mucosal thickening of maxillary sinuses was measured on CBCT coronal reconstruction. It was defined by presence of mucosa around the maxillary sinus walls over 3 mm of height ( Fig. 3 ). 19 20 Moreover, the side of the region affected by the mucosal thickening (right, left, or both sides) was recorded.

#### Sex and Age

Individual's sex and age were also tabulated in a spreadsheet for the analysis of a possible association with the presence of CB.

Examiners evaluated no more than 20 CBCT exams per day, and an interval of at least 72 hours between sessions was established to prevent visual fatigue. Imaging tools—such as brightness, contrast, and zoom—could be applied as in a clinical scenario. 50 days after the evaluations ended, 30% of the sample (89 randomly selected exams) was reassessed for consistency.

### Data Analyses

Statistical analyses were performed using the SPSS 25.0 software (SPSS Inc.), with the significance level set at 5% ( *p* < 0.05) *.* First, multinomial logistic regression was used to determine whether any of the evaluated factors (sex, age, NSD, and maxillary sinus mucosal thickening) could predict the presence of CB. Then, specific associations, such as the type of CB (upper, middle, or lower), pneumatization side, NSD, mucosal thickening of the maxillary sinus, sex, and age, were assessed by chi-squared or Fisher's exact tests. Additionally, the percentage of correct answers between the evaluation and re-evaluation of 30% of the sample was calculated to assess the consistency of the results.

## Results

Considering all patients (297), 132 (44.4%) had at least one CB. When considering all evaluated turbinates (1782), 208 (11.7%) CBs were identified in the investigated population. Furthermore, of all the CBs found, 113 (54.32%) were from females and 95 (45.98%) from males. In general, multinomial logistic regression showed that sex, age, presence of NSD or maxillary sinus mucosal thickening could not be considered as predictor of the presence of CB ( *p* > 0.05), as observed in Table 1 .

**Table 1:** Results of multinomial logistic regression

Effect	-2 log likelihood of reduced model	Likelihood ratio tests		
		Chi-squared	df	*p* -value*
**Intercept**	111.509	0.000	0	
**Sex**	111.762	0.252	1	0.616
**Age**	119.028	7.519	6	0.276
**Presence of nasal septum deviation**	111.523	0.014	1	0.906
**Presence of mucosal thickening of the maxillary sinus**	111.691	0.181	1	0.670

**Note:** *According to multinomial logistic regression.

There were 40 CBs identified in the upper nasal turbinates (upper: 6.7%; CBs: 19.2%), 158 CBs were found in the middle (middle: 26.6%; CBs: 76.0%), and 10 CB were identified in the lower nasal turbinates (lower: 1.7%; CBs: 4.8%). The presence of CBs in the middle turbinates was significantly higher ( *p* < 0.0001) than in the others, as shown in Table 2 . Also, there was no association between CB and the side of pneumatization, as indicated in Table 3 .

**Table 2:** Concha bullosa prevalence according to the turbinate evaluated

CB	Absent (%)	Present (%)
Upper turbinates	554 (93.3)	40 (6.7)
Middle turbinates	436 (73.4)	158 (26.6)
Lower turbinates	584 (98.3)	10 (1.7)

**Abbreviation:** CB, concha bullosa. **Note:** Statistical higher prevalence of concha bullosa on middle turbinates, according to the chi-square test ( *p* < 0.0001).

**Table 3:** Concha bullosa prevalence according to the side of pneumatization (right/left)

CB	Right turbinate	Left turbinate
Upper	23 (57.5)	17 (42.5)
Middle	79 (50)	79 (50)
Lower	4 (40)	6 (60)

**Abbreviation:** CB, concha bullosa. **Note:** Without significant differences according to the chi-square test ( *p* = 0.542).

Table 4 shows an association with NSD, as the middle-left CB was associated with a higher prevalence ( *p* = 0.028). We found 29 middle left CBs in the presence of NSD and, in these cases, the number of those deviated to the right (n = 22, 79.31%) was significantly higher than to the left (n = 7, 20.69%; *p* = 0.009, chi-square test).

**Table 4:** Distribution of concha bullosa according to the condition of the nasal septum

CB position		Nasal septum deviation (%)		Total	*p* -value
		Ausence	Presence		
**Upper right**	Ausence	198 (72.26)	76 (27.74)	274 (100)	0.633**
	Presence	18 (78.26)	5 (21.74)	23 (100)	
**Upper left**	Ausence	201 (71.78)	79 (28.22)	280 (100)	0.170**
	Presence	15 (88.23)	2 (11.77)	17 (100)	
**Middle right**	Ausence	161 (73.85)	57 (26.15)	218 (100)	0.469*
	Presence	55 (69.62)	24 (30.38)	79 (100)	
**Middle left**	Ausence	166 (76.15)	52 (23.85)	218 (100)	0.028*
	Presence	50 (63.29)	29 (36.71)	79 (100)	
**Inferior right**	Ausence	213 (72.7)	80 (27.3)	293 (100)	1.000**
	Presence	3 (75)	1 (25)	4 (100)	
**Inferior left**	Ausence	210 (72.16)	81 (27.84)	291 (100)	0.194**
	Presence	6 (100)	0 (0)	6 (100)	

**Abbreviation:** CB, concha bullosa. **Note:** Values in bold indicate statistically significant association between the factors, according to the chi-squared test ( *p* = 0.028). * According to the chi-squared test. ** According to Fisher's exact test.

Tables 5 and 6 show that, while no association was found between the presence of CB and mucosal thickening in the right maxillary sinus, there was an association between CB and mucosal thickening in the left maxillary sinus (> 3 mm in height; *p* = 0.028). However, this statistical difference must be carefully interpreted, since the number of lower left CBs was small (n = 6). Moreover, there was no association ( *p* > 0.05) between the presence of CB and the individual's sex or age, as indicated in Tables 7 and 8 , respectively.

**Table 5:** Distribution of concha bullosa according to the condition of the right maxillary sinus

CB position		Mucosal thickening of the right maxillary sinus (%)		Total	*p* -value
		Ausence	Presence		
**Upper right**	Ausence	178 (64.96)	96 (35.04)	274 (100)	0.057**
	Presence	20 (86.95)	3 (13.05)	23 (100)	
**Middle right**	Ausence	145 (66.52)	73 (33.48)	218 (100)	0.926*
	Presence	53 (67.09)	26 (32.91)	79 (100)	
**Lower right**	Ausence	196 (66.89)	97 (33.11)	293 (100)	0.603**
	Presence	2 (50)	2 (50)	4 (100)	

**Abbreviation:** CB, concha bullosa. **Note:** * According to the chi-squared test. ** According to Fisher's exact test.

**Table 6:** Distribution of concha bullosa according to the condition of the left maxillary sinus

CB position		Mucosal thickening of the left maxillary sinus (%)		Total	*p* -value
		Ausence	Presence		
**Upper left**	Ausence	177 (63.21)	103 (36.79)	280 (100)	0.716*
	Presence	10 (58.82)	7 (41.18)	17 (100)	
**Middle left**	Ausence	144 (66.05)	74 (33.95)	218 (100)	0.067*
	Presence	43 (54.43)	36 (45.57)	79 (100)	
**Lower left**	Ausence	186 (63.92)	105 (36.08)	291 (100)	0.028**
	Presence	1 (16.67)	5 (83.33)	6 (100)	

**Abbreviation:** CB, concha bullosa. **Note:** Values in bold indicate statistically significant association between the factors, according to the Fisher's exact test ( *p* = 0.028). * According to the chi-squared test. ** According to Fisher's exact test.

**Table 7:** Concha bullosa distribution according to the individual's sex

CB position		Sex (%)		Total	*p* -value
		Female	Male		
**Upper right**	Ausence	142 (51.82)	132 (48.18)	274	0.974*
	Presence	12 (52.17)	11 (47.83)	23	
**Upper left**	Ausence	144 (51.43)	136 (48.57)	280	0.554*
	Presence	10 (58.82)	7 (41.18)	17	
**Middle right**	Ausence	110 (50.45)	108 (49.55)	218	0.425*
	Presence	44 (55.7)	35 (44.3)	79	
**Middle left**	Ausence	113 (51.83)	105 (48.17)	218	0.992*
	Presence	41 (51.9)	38 (48.1)	79	
**Lower right**	Ausence	151 (51.54)	142 (48.46)	293	0.351**
	Presence	3 (75)	1 (25)	4	
**Lower left**	Ausence	151 (51.89)	140 (48.11)	291	0.927**
	Presence	3 (50)	3 (50)	6	

**Abbreviation:** CB, concha bullosa. **Note:** * According to the chi-squared test. ** According to Fisher's exact test.

**Table 8:** Concha bullosa prevalence according to individual's age

CB position		18-20	21-30	31-40	41-50	51-60	> 60	*p* -value
**Upper**	Ausence	76 (13.7)	244 (44.0)	101 (18.2)	85 (15.3)	36 (6.5)	12 (2.2)	0.332**
	Presence	4 (10.0)	14 (35.0)	11 (27.5)	5 (12.5)	4 (10.0)	2 (5.0)	
**Middle**	Ausence	57 (13.1)	192 (44.0)	83 (19.0)	71 (16.3)	22 (5.0)	11 (2.5)	0.113*
	Presence	23 (14.6)	66 (41.8)	29 (18.4)	19 (12.0)	18 (11.4)	3 (1.9)	
**Lower**	Ausence	78 (13.4)	251 (43.0)	112 (19.2)	89 (15.2)	40 (6.8)	14 (2.4)	0.474**
	Presence	2 (20.0)	7 (70.0)	0 (0.0)	1 (10.0)	0 (0.0)	0 (0.0)	

**Abbreviation:** CB, concha bullosa. **Note:** * According to the chi-squared test. ** According to Fisher's exact test.

Finally, the percentage of correct answers between the evaluation and re-evaluation of the investigated structures or anatomical variations showed excellent value, as presented in Table 9 , demonstrating consistent assessments.

**Table 9:** Correct answers between evaluation and revaluation according to the anatomical structure or anatomical variation assessed

Anatomical structure/variation	Percentage (%)
**Turbinates**	
Upper right	94
Upper left	91
Middle right	91
Middle left	91
Lower right	98
Lower left	98
**Nasal septum deviation**	85
**Mucosal thickening**	
Right maxillary sinus	90
Left maxillary sinus	91

## Discussion

Since CB may be associated with symptomatic patients and surgical interventions, it is necessary to comprehend the prevalence of this anatomical variation in different populations. To our knowledge, the present research is the first to evaluate the presence of CB in all nasal turbinates (upper, middle, and lower), since the majority of studies evaluated only the middle one. 10 13 Additionally, possible association between CB and side of pneumatization, NSD, mucosal thickening of the maxillary sinus, sex, and age were investigated.

The prevalence values (11.7%) were relatively lower compared to previous studies (21–53%). 8 9 In addition to the differences between the investigated populations, three other factors may explain this divergence in the results. Firstly, most studies only evaluated the CB prevalence in the middle nasal turbinates, not assessing pneumatization in the upper and/or lower areas. 8 10 11 When the prevalence is considered only in the middle turbinate, our results are closer to previous findings, with a percentage of 26.6%. Furthermore, the evaluation methods employed differed. While the present study used CBCT exams, others used CT or surgical evaluations. 4 9 13 Finally, the CB classification may vary. The definition used here was by Stallman et al., who classified the nasal turbinate as bullosa when 50% of its vertical height is pneumatized. 9 10 However, considering all the CBCT exams assessed, 44% of them had at least one CB, a percentage similar to that found in a previous research using CT exams, which reported 35%. 21

The presence of CB in the middle turbinates was significantly higher when compared to the others. This result agrees with those of previous studies. 22 23 Furthermore, only 10 cases of lower CB were found, corresponding to 4.8%. This finding is also consistent with the consulted literature, since pneumatization of the lower nasal turbinate is uncommon. 23

Regarding the investigated variables, we have found a significant association between the presence of CB and NSD. Although it is hypothesized that the etiology of NSD is not associated with the pneumatization of nasal turbinates, but with other factors, such as genetic predisposition or trauma, a strong association between these anatomical variations has been previously found. 10 11 24 Two possible theories have been proposed: the first suggests that, as the CB increases in size due to its pneumatization, the nasal septum deviates in the opposite direction to the turbinate growth in an attempt to unblock the nasal cavity's drainage pathway. 10 The second theory posits that, in the presence of NSD, the turbinates begin to pneumatize, filling the space created by the misalignment in an attempt to preserve the respiratory system's function. 11 The results showed a high prevalence of left middle CBs when NSD was present. Considering that in 79.31% of these cases the deviation was to the right side, the opposite direction of the CB, our study corroborates this association. However, due to the observational design of the study, it is not possible to identify what has happened first.

Previously, Bhandary et al. showed that the presence of CB could predispose individuals to a sinus disease, defined as partial or complete opacification observed on CT exams. 8 However, according to our results, there is no association between CB and mucosal thickening of the maxillary sinus, regardless of the side (right or left maxillary sinus) assessed. A previous research that investigated its relationship with accessory maxillary ostium, as well as pathological mucosal thickening of the maxillary sinus on CBCT images, showed similar results, as no correlation was found among these three variables. 25 Considering patients may be predisposed to a higher incidence of allergies, including sinusitis, during the winter season, this divergence in the results may be a consequence of a seasonal bias. 26

It is common to use CT as a complementary exam to assess the nasal-sinus complex. 9 13 However, CBCT exams offer some advantages compared to the multidetector modality, as they reduce the patient's exposure to X-rays, have a faster scanning time, provide better spatial resolution, and have a lower machine cost. 27 Furthermore, these exams, which are commonly requested in dental practice, can be acquired with large FOVs, which allows the evaluation of a wider region, including structures of the nasal-sinus complex. Therefore, CBCT can be an advantageous alternative compared to CT in this clinical scenario.

In symptomatic cases of CB, surgical management may be necessary to restore patients' health. 16 Among the techniques, resection, medial marsupialization, and lateral marsupialization can be considered, with the latter showing excellent results and no postsurgical complications, according to the literature. 28 Given the positive prognosis of surgical intervention in symptomatic patients, identifying CB as a cause of respiratory dysfunction is extremely important.

The results of this study showed that the percentage of correct answers between the evaluation and reevaluation of the investigated structures and anatomical variations was excellent, demonstrating that consistency in the assessments. However, our findings cannot be extrapolated to other populations. Therefore, further research involving CB and individuals from different populations is encouraged. Furthermore, other anatomical variations of the sinus complex (such as Haller cells, agger nasi, paradoxical turbinates, and pneumatization of the unciform process) and potential associations among them should also be investigated.

## Conclusion

Almost half of the CBCT exams had at least one CB, with the middle turbinate being more affected. There seems to be an association between middle CB and the presence of NSD. Furthermore, no association was found between CB, side of pneumatization, mucosal thickening of the maxillary sinus, and individual's sex or age.
